# Assessment of early damage of endometrium after artificial abortion by shear wave elastography

**DOI:** 10.1186/s13244-020-0841-4

**Published:** 2020-03-04

**Authors:** Yan Jiao, Nianyu Xue, Chunpeng Zou, Xujuan Shui, Hongqing Wang, Chunhong Hu

**Affiliations:** 1 Department of Radiology, The First Affiliated Hospital of Soochow University, No. 188 Shizi Street, Suzhou, 215006 China; 2 Obstetrics and Gynecology Ultrasonic Department, Wenzhou Peoples’ Hospital, Wenzhou, 325000 China; 3 Department of Diagnostic Ultrasonography, Ningbo First Hospital, Ningbo, 315010 China; 4 Department of Diagnostic Ultrasonography, The Second Affiliated Hospital and Yuying Children’s Hospital of Wenzhou Medical University, Wenzhou, 325027 China; 5 Department of Radiology, The First Affiliated Hospital of Wenzhou Medical University, Wenzhou, 325000 China

**Keywords:** Shear wave elastography, Endometrium, Elasticity, Artificial abortion, Young’s modulus

## Abstract

**Objectives:**

This study aimed to investigate the application of shear wave elastography (SWE) in the early damage detection through assessing the endometrial elasticity after artificial abortion.

**Methods:**

A total of nulliparous women (20–30 years) who received ultrasonography in our hospital were recruited between January 2017 and December 2017. These women were divided into normal control group (NC; *n* = 65), after once artificial abortion group (AOAA; *n* = 68), after twice artificial abortion group (ATAA; *n* = 61), and after three times or more (range, 3–6) artificial abortion group (ATTMAA; *n* = 60). SWE was performed to evaluate the endometrium; Young’s modulus of the endometrium was determined and then the endometrial thickness was measured.

**Results:**

Young’s modulus of the endometrium increased in the order of NC group, AOAA group, ATAA group, and ATTMAA group, and Young’s modulus increased with the increase in the number of artificial abortions (*p* < 0.05). The endometrial thickness in the ATTMAA group was significantly lower than in the NC group, AOAA group, and ATAA group (*p* < 0.05), but there was no marked difference among the NC group, AOAA group, and ATAA group (*p* > 0.05).

**Conclusions:**

SWE increases with increasing number of abortions, which may indicate the damage that is done to the endometrium earlier than measurement of the endometrial thickness do.

## Key points

- SWE is effective to detect the endometrial elasticity.
- Assessment of endometrial elasticity by SWE may be a better indication in detecting early endometrial damage than measurement of the endometrial thickness.

## Introduction

Artificial abortion is common due to the no pregnancy intention, limited medical resources, and poor reproductive health education [1–3]. In recent years, artificial abortion still has a high prevalence and has become an important public health problem [4–6]. Artificial abortion may cause mechanical damage to the endometrium, which increases the risk for some complications such as reproductive tract infection, intrauterine adhesions, and secondary infertility. This significantly affects the mental health and quality of life of women [7–9]. The endometrial recovery after artificial abortion is of great importance. In available studies, some clinical characteristics (such as the time of sustained vaginal bleeding, menstrual blood loss, and time to menstrual cycle regularity) and endometrial thickness are employed to evaluate and monitor the endometrial recovery [10, 11]. However, these factors that indirectly reflect the endometrial recovery and the endometrial thickness may not reflect the endometrial elasticity, which limit their applications in clinical practice.

Shear wave elastography (SWE) is a technique used to evaluate the elasticity of living tissue. It is a real-time, non-invasive, and quantitative technique. In SWE, the probe emits safe acoustic radiation pulses, which can focus on tissues at different depths and continuously induce the tissue particles to vibrate and produce transverse shear wave which is then accurately measured [12]. SWE has been used to evaluate the elasticity of a variety of normal and/or injured tissues [13–15].

Manchanda et al. applied SWE to study the elasticity of normal uterus, and they found no significant difference in the mean endometrial elasticity among women in different menstrual stages or different age groups and no age-related difference in the mean cervical elasticity [16]. Ono et al. revealed that the cervical hardness during pregnancy was negatively related to the gestational age [17]. Zhang et al. found that the myometrial hardness in patients with adenomyosis and leiomyomas was greater than that of the normal myometrium [18]. Currently, little is known about the use of SWE in the detection of elasticity of diseased endometrium. This study aimed to investigate the application of SWE in the assessment of endometrial elasticity after artificial abortion.

## Materials and methods

### Subjects

A total of 254 nulliparous women (20–30 years) who received gynecological ultrasonography in our hospital were reviewed between January 2017 and December 2017. Their menstrual cycle ranged from 26 to 30 days. According to the number of artificial abortion, these women were divided into normal control group (NC; *n* = 65; no abortion; mean age 24.39 ± 4.26 years), after once artificial abortion group (AOAA; *n* = 68; mean age, 26.01 ± 3.85 years), after twice artificial abortion group (ATAA; *n* = 61; mean age 25.56 ± 4.53 years), and after three times or more (range 3–6) artificial abortion group (ATTMAA; *n* = 60; mean age, 24.91 ± 4.11 years). Exclusion criteria were as follows: (1) pathology of the uterus, ovary, and fallopian tube; (2) use of drugs that may affect the endometrium (such as estrogen and progesterone) within 1 month before ultrasonography; (3) presence of uterine effusion, intrauterine adhesions, and intrauterine residual or normal menstrual cycle within 3 months after the last artificial abortion. The study was conducted in accordance with the Committee for Human Research at our institution and followed all regulations. Informed consent was obtained before the study.

### Instruments and methods

Supersonic Imagine AixPlorer ultrasonic instrument with SWE (Shear Wave^TM^) was used for the detection of endometrial elasticity. The SE12-3 probe (frequency 3–12 MHz) applicable for SWE was used.

**Fig. 1:**
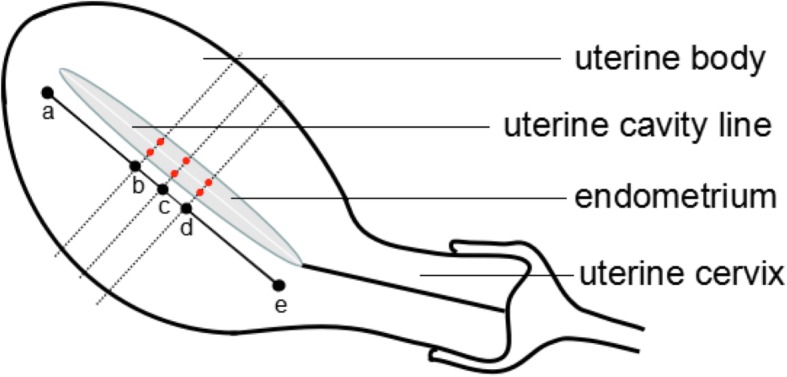
Sites of endometrium for the measurement of endometrial elasticity by SWE. Notes: Line ae and uterine cavity line are parallel and have the same length; the three dotted lines are vertical to the line ae and endometrial line; point c is the middle point between point a and point e; point b localizes 0.5 cm away from the point c (close to a); point d localizes 0.5 cm away from the point c (close to e). Red dots represent the six sampling sites for SWE.

For subjects, SWE of the endometrium was performed by the same sonographer. Meanwhile the examination was performed 3 months after the last abortion. The endometrial elasticity was evaluated in the ovulation stage (days 14–16 of menstrual cycle; maximal diameter of follicles, 18–22 mm). In brief, women lied in a supine position, and routine abdominal ultrasonography was performed to explore the pelvic organs. Then, transvaginal two-dimensional ultrasonography was done to observe the shape, outline, and internal structures of the uterus after emptying the bladder. Thereafter, transvaginal ultrasonography was done at multiple views to evaluate the symmetry of the uterus, possible presence of uterine effusion and other lesions, and assess the endometrial echoes and thickness as well as the borderline between the endometrium and myometrium. The endometrial thickness was measured at the maximal longitudinal section and recorded. Measurement was done at the middle point between the fundus of uterus and cervix. Then, SWE of the endometrium was performed with the SWE mode. The red, green, and blue colors represent high, intermediate, and low Young’s modulus, respectively. When the images became stable, the images were frozen, and quantification was done with the Q-BOX system. Young’s modulus of the anterior and posterior endometrium was measured. The diameter of region of interest (ROI) was 2 mm, and the distance between ROI and probe was 2–4 cm. The ROI was set at three sites of the anterior endometrium and three sites of the posterior endometrium (middle point between the fundus of uterus and cervix, 0.5 cm away from the middle point [close to the fundus of uterus] and 0.5 cm away from the middle point [close to the cervix]) (Fig. 1). All these ROIs localized at the middle points between endometrium-myometrium boundary line and uterine cavity line. The mean, maximal, and minimal Young’s moduli were automatically calculated by the Q-BOX system. The mean Young’s modulus of six sites was determined. At the same time, blood was collected on the day of ultrasonography, and the serum levels of estradiol and progesterone were detected and recorded.

### Statistical analysis

Data were expressed as mean ± standard deviation (SD). Statistical analyses were performed using the statistical package for social software version 22 (SPSS Inc., Chicago, IL, USA). Comparisons of quantitative data were done using one-way analysis of variance (ANOVA) followed by post hoc least significant difference (LSD) test. A value of *p* < 0.05 was considered statistically significant.

## Results

**Fig. 2:**
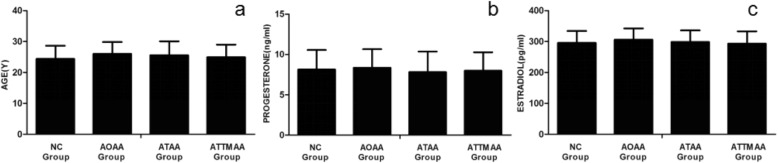
Age, progesterone, and estradiol in the NC group, AOAA group, ATAA group, and ATTMAA group. **a** age; **b** progesterone; **c** estradiol

There were no marked differences in age and serum levels of progesterone and estradiol among the NC group, AOAA group, ATAA group, and ATTMAA group (*P* > 0.05) (Table 1; Fig. 2).

**Table 1:** Parameters in different groups

Group	*n*	Age (years)	Estradiol (ng/ml)	Progesterone (pg/ml)	Endometrial thickness (mm)	Young’s modulus (kPa)
NC	65	24.39 ± 4.26	8.13 ± 2.42	295.89 ± 38.24	11.25 ± 2.81	10.17 ± 3.81
AOAA	68	26.01 ± 3.85	8.35 ± 2.31	306.16 ± 36.37	12.03 ± 2.59	17.01 ± 4.63^*^
ATAA	61	25.56 ± 4.53	7.80 ± 2.55	298.35 ± 37.66	11.57 ± 2.75	23.35 ± 4.92^*#^
ATTMAA	60	24.91 ± 4.11	7.98 ± 2.29	293.13 ± 40.08	8.25 ± 2.06^*#Δ^	32.17 ± 5.38^*#Δ^
*P*_AOAA vs NC_		0.07	0.59	0.11	0.10	0.00
*P*_ATAA vs NC_		0.14	0.46	0.72	0.52	0.00
*P*_ATTMAA vs NC_		0.49	0.72	0.69	0.00	0.00
*P*_ATAA vs AOAA_		0.55	0.20	0.23	0.33	0.00
*P*_ATTMAA vs AOAA_		0.12	0.37	0.06	0.00	0.00
*P*_ATTMAA vs ATAA_		0.41	0.68	0.46	0.00	0.00

^*^p < 0.05 vs NC group; ^#^*p* < 0.05 vs AOAA group; ^Δ^*p* < 0.05 vs ATAA group

**Fig. 3:**
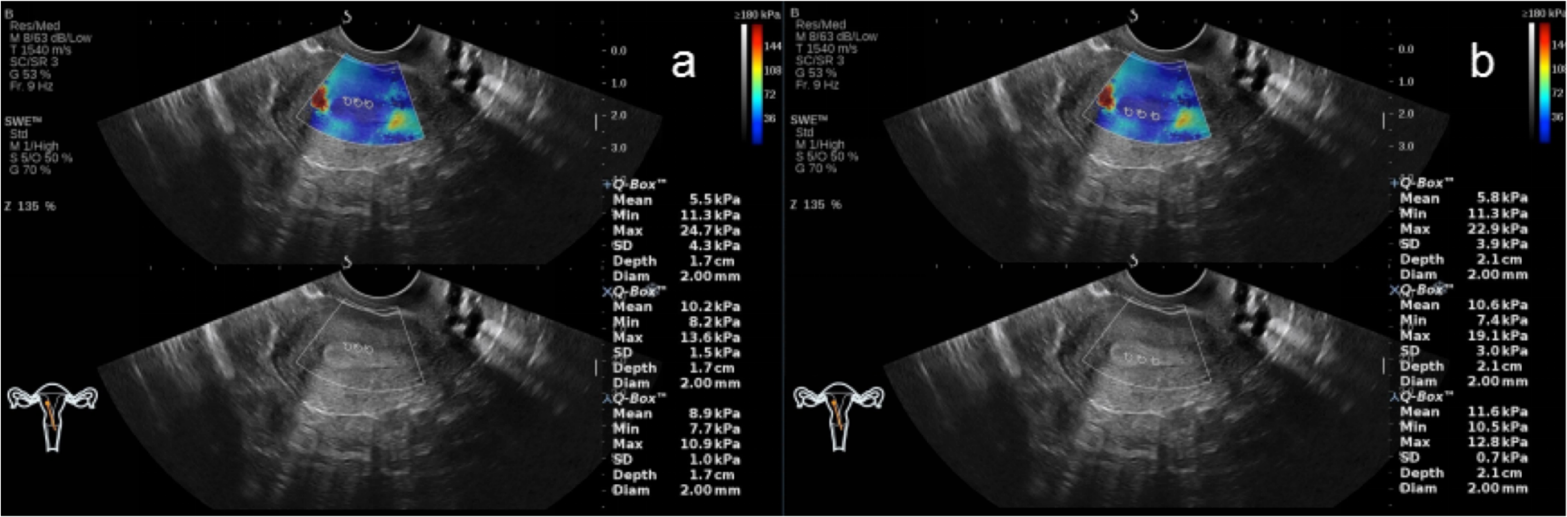
Representative images of a woman in the NC group. **a** Young’s modulus at the three sites of the anterior endometrial wall was 5.5, 10.2, and 8.9 kPa, respectively; **b** Young’s modulus at the three sites of the posterior endometrial wall was 5.8, 10.6, and 11.6 kPa, respectively. The mean Young’s modulus of the six sampling sites was 8.8 kPa

**Fig. 4:**
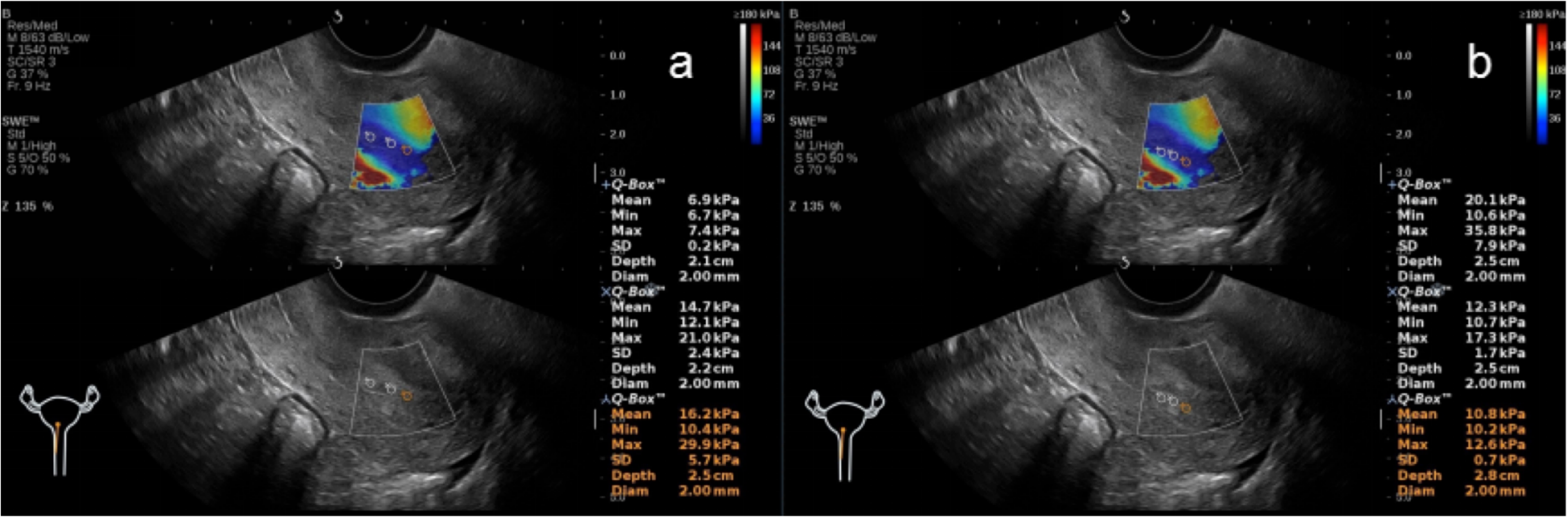
Representative images of a woman in the AOAA group. **a** Young’s modulus at the three sites of the anterior endometrial wall was 6.9, 14.7, and 16.2 kPa, respectively; **b** Young’s modulus at the three sites of the posterior endometrial wall was 20.1, 12.3, and 10.8 kPa, respectively. The mean Young’s modulus of the six sampling sites was 13.5 kPa

**Fig. 5:**
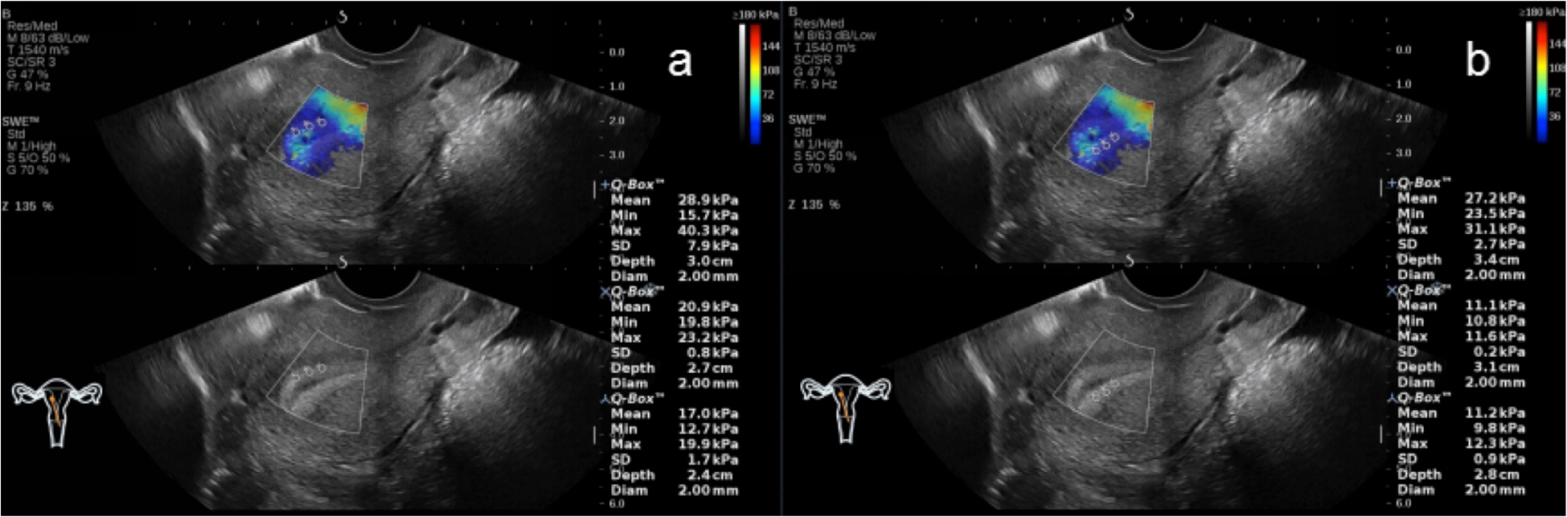
Representative images of a woman in the ATAA group. **a** Young’s modulus at the three sites of the anterior endometrial wall was 28.9, 20.9, and 17.0 kPa, respectively; **b** Young’s modulus at the three sites of the posterior endometrial wall was 27.2, 11.1, and 11.2 kPa, respectively. The mean Young’s modulus of the six sampling sites was 19.4 kPa

**Fig. 6:**
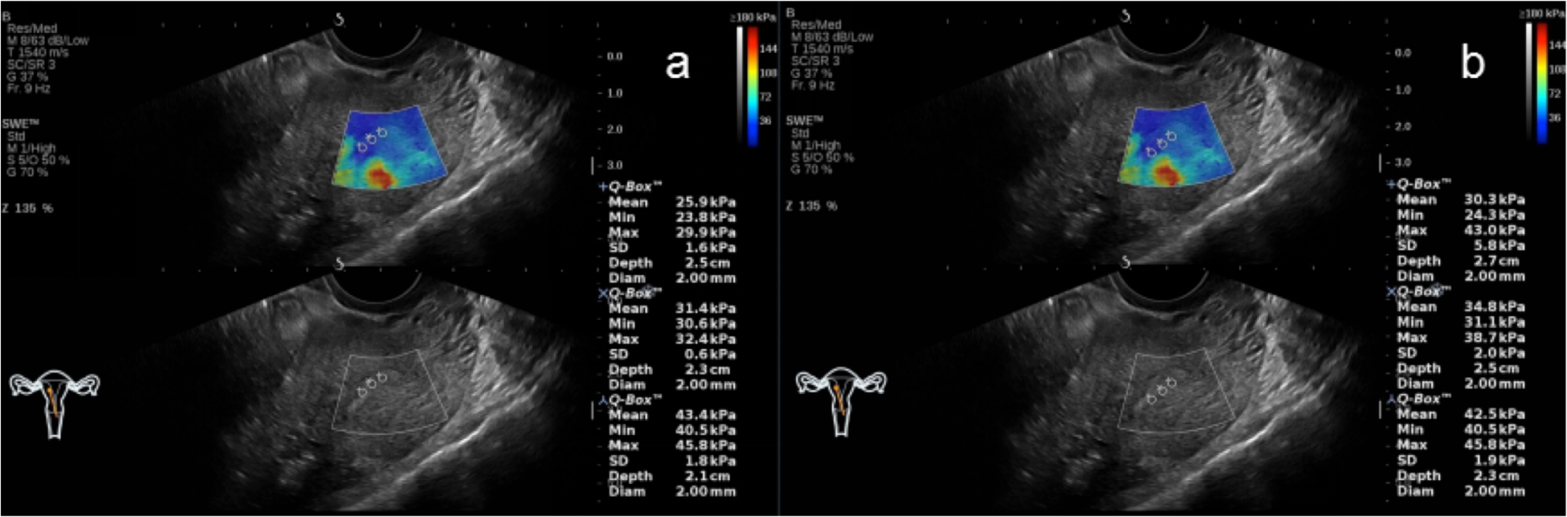
Representative images of a woman in the ATTMAA group. **a** Young’s modulus at the three sites of the anterior endometrial wall was 25.9, 31.4, and 43.4 kPa, respectively; **b** Young’s modulus at the three sites of the posterior endometrial wall was 30.3, 34.8, and 42.5 kPa, respectively. The mean Young’s modulus of the six sampling sites was 34.7 kPa

**Fig. 7:**
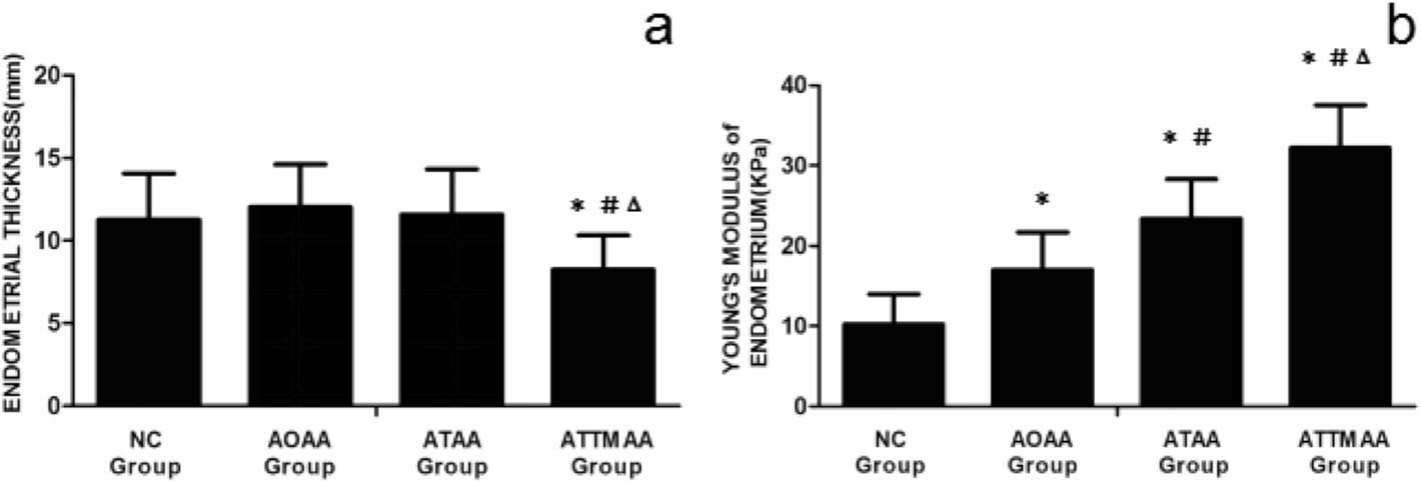
Young’s modulus endometrium and endometrial thickness in the NC group, AOAA group, ATAA group, and ATTMAA group. **a** Endometrial thickness in the NC group, AOAA group, ATAA group, and ATTMAA group. **b** Young’s modulus of the endometrium in the NC group, AOAA group, ATAA group, and ATTMAA group. *p < 0.05 vs NC group. ^#^*p* < 0.05 vs AOAA group. ^Δ^*p* < 0.05 vs ATAA group

In the NC group, AOAA group, ATAA group, and ATTMAA group, the endometrial thickness was 11.25 ± 2.81 mm, 12.03 ± 2.59 mm, 11.57 ± 2.75 mm, and 8.25 ± 2.06 mm, respectively. The endometrial thickness in the ATTMAA group was significantly lower than in the NC group, AOAA group, and ATAA group (*p* < 0.05), but there was no marked difference among the NC group, AOAA group, and ATAA group (*p* > 0.05). In the NC group, AOAA group, ATAA group, and ATTMAA group, Young’s modulus was 10.17 ± 3.81 kPa, 17.01 ± 4.63 kPa, 23.35 ± 4.92 kPa, and 32.17 ± 5.38 kPa, respectively. Young’s modulus was significantly different between any two groups (*p* < 0.05) (Figs. 3, 4, 5 and 6). Young’s modulus increased with the increase in the number of artificial abortion (Table 1; Fig. 7).

## Discussion

In the present study, the endometrial thickness and elasticity were assessed in post-abortion patients and normal subjects. Results showed that endometrial thickness in the ATTMAA group was significantly lower than in the NC group, AOAA group, and ATAA group, but there was no marked difference among the AOAA group, ATAA group, and NC group. In addition, Young’s modulus increased significantly in the order of NC group, AOAA group, ATAA group, and ATTMAA group. These findings indicate that the endometrial elasticity reduces after repeated artificial abortion in the ATTMAA group; although the endometrial thickness in the AOAA group and ATAA group remains unchanged, the physical characteristics of the endometrium change significantly (elasticity reduces and stiffness increases), which was more evident in the TAA group than in the AOAA group.

Some investigators found the mechanical damage to the endometrium could reduce the endometrial glandular epithelium and increase the collagen fibers in animal model of artificial abortion [19], leading to the increased proportion of collagen fibers in the endometrium. There is evidence showing that the increased collagen fibers in the organ may significantly compromise the elasticity of this organ and increases its stiffness [20, 21]. Thus, on the basis of our findings, we speculate that the mechanical damage to the endometrium after artificial abortion may cause hyperplasia of collagen fibers in the endometrium and thereafter increase their amount, which leads to the increase in the endometrial stiffness [22–24]. Moreover, the endometrial stiffness increases with the increase in the number of artificial abortion. The pathological changes in the endometrium are consistent with the change in the endometrial stiffness. Recent studies have shown that SWE can be used to evaluate the elasticity of normal myometrium, intima, and cervix and that SWE is a promising tool for the uterine assessment and helpful for the diagnosis of various uterine diseases [16]. Available findings together with our results indicate that SWE may be used to display the early damage to the endometrium when it is invisible on conventional ultrasonography and 2D measurements. Taking this research as an opportunity, we can carry out more systematic studies on the application of SWE in the assessment of endometrium after artificial abortion. This is a direction of our future investigations.

There were limitations in this study. The uterus is three-dimensional, and current techniques cannot sample the endometrium at different sites simultaneously. Thus, the middle points of the anterior and posterior walls of the uterus were selected. We believe that the development of medical technology will improve the measurement of endometrial elasticity. However, our findings provide valuable information for the clinical assessment of endometrial damage after artificial abortion, which is helpful for the diagnosis and management of endometrial complications after abortion.

Taken together, SWE is effective to detect the endometrial elasticity after artificial abortion, which seems to indicate the assessment of physical characteristics and pathological changes of the endometrium. The changes in the endometrium after artificial abortion may occur preceding the clinical manifestations and the alteration of endometrial thickness. Thus, the endometrial elasticity determined by SWE may provide important information for the assessment of endometrial recovery.

## Data Availability

The datasets used and/or analyzed in the present study are available from the corresponding author on reasonable request.

## Abbreviations

AOAA: After once artificial abortion
ATAA: After twice artificial abortion
ATTMAA: After three times or more artificial abortion
ROI: Region of interest
SD: Standard deviation
SWE: Shear wave elastography
